# Recent Experiences of Intussusception

**Published:** 1928

**Authors:** Hubert Chitty

**Affiliations:** Surgeon to the Bristol Royal Infirmary and Bristol Children's and Orthopœdic Hospitals


### RECENT EXPERIENCES OF INTUSSUSCEPTION.

BY

HUBERT CHITTY, M.S., F.R.C.S.,

*Surgeon to the Bristol Royal Infirmary and Bristol Children's and Orthopædic Hospitals.*

---

SINCE 1919 I have operated on thirty-six patients for intussusception; at the Infirmary twenty cases, at the Children's Hospital fourteen cases (since 1922), and two cases in my private practice during the same period.

Of these, two were irreducible gangrenous cases, one with a four days' history, one of thirty-six hours only. I resected the gangrenous gut in both cases, and both died. Of the thirty-four reducible cases one, a patient with an intussusception of the ileo-colic type, with a three days' history, died, the remaining thirty-three recovered. The mortality of reducible cases was, therefore, just under 3 per cent., that of all cases combined rather less than 9 per cent.

Twenty-six patients were under one year of age, the youngest one month. Five were between one and two years old. One was aged six years, and two were aged eight years. In one case the age was not specified.

Most of them were operated upon within twenty-four hours of the onset of their symptoms, earliest two hours; but I have had recoveries after two days, three days, four days and two weeks. I shall refer to these later.

[One child had a recurrence after three months. One child who had been operated upon for hypertrophic stenosis of the pylorus was readmitted with an intussusception the day after his discharge from hospital. One was found to be suffering from tuberculous peritonitis in addition to its intussusception. In two cases reduction was achieved by rectal injections and confirmed by operation. Most of the cases showed typical histories, signs and symptoms.

It may not be amiss to run rapidly over the typical features of a text-book case. A previously healthy, breast-fed baby is suddenly seized with an acute attack of colic. It screams with pain, draws up its legs, turns pale, and frequently vomits or passes a motion. It then commonly falls asleep again, only to be awakened a short while later by a similar attack of colic. Should the bowels act a second time only blood and slime are passed. This motion does not smell offensive. Careful palpation during a peaceful interlude will generally reveal an absence of the normal resistance in the right iliac fossa. A tumour, curved in its long axis, may generally be felt. Its consistence varies, so that at one moment it is readily palpable, while at the next it may have apparently disappeared. A rise of temperature is not unusual, though in cases showing collapse it may become subnormal.

Emptiness in the right iliac fossa is due to the fact that most intussusceptions start in this region, so that very early the cæcum is turned inside out, or rather outside in, thus causing it to disappear from its normal position, its place being taken by much less resistant small intestine.

When tucked up under the ribs, the tumour may be very difficult to detect. It hardens as a wave of peristalsis passes along it, and becomes soft as this passes away. Its shape is due to the fact that, as the gut is invaginated, it drags with it the mesentery ; hence the mesenteric border of the intussuscepted gut is shorter than its antimesenteric aspect. As the mesentery is dragged in its veins are compressed more readily than its arteries, hence the bowel becomes œdematous and its lumen occluded, while the congested mucous membrane exudes mucus and blood.

In ileo-colic cases the intussusception begins in the last few inches of the small intestine, and therefore has very soon to pass through the ileo-cæcal valve. Here it is apt to be tightly gripped. Typical signs, therefore, appear early and are very acute. When an intussusception commences in the cæcum or colon, however, a long mesocolon is found to be present, and it is possible for a considerable amount of invagination to take place into this more capacious gut before complete obstruction or even exudation of blood occurs. The diagnosis is, therefore, apt to be delayed. All the late cases in this series which ended in recovery were of this type. Cases are on record in which symptoms have been present for many weeks, in spite of which reduction has been possible and eventual recovery has taken place. On the other hand, such a child may at any time develop acute symptoms and the result may prove fatal.

I propose to give a short account of a few of the less usual cases in this series.

*Peculiar site of Intussusception.*

**Case 1.**—A child aged two years was admitted with a history of acute abdominal pain, colicky in nature, of two days' duration. During the last day blood and mucus had been passed per rectum. There was no emptiness in the right iliac fossa, but in the left iliac fossa a definite tumour could be felt. This was also readily palpable per rectum. A rectal injection was given, and the child anæsthetised, when the tumour was found to have disappeared. In spite of this, it was deemed safer to make an exploratory incision and the sigmoid flexure was pulled up into the wound. In this was found a definite ring of œdematous gut where the head of the intussusception must have existed.

*Long duration of Intussusception.*

**Case 2.**—A child of six years was admitted with a *three* days' history, typical in every respect. A tumour could be felt, both abdominally and per rectum, extending into the sigmoid flexure. In spite of the duration of symptoms, it was easily reduced, and its apex was found to be the caput cæci.

**Case 3.**—A child aged eight was admitted into the Bristol Royal Infirmary with the following history. Early that morning she had been seized with violent colic, which recurred several times, but had passed away by the time she was brought into hospital, and the child appeared perfectly comfortable. Neither blood nor mucus was passed. When she was examined by the resident she was found to have a temperature of 101°, and some resistance was felt beneath the upper part of the right rectus. When I came down that evening to operate on another patient I was asked to see her. She was sleeping peacefully, but I was able to feel a tumour in the right upper epigastrium. At one moment it was quite definite; at the next one doubted if it was there. Palpation caused not the slightest pain. In view of some of my previous cases, I made a diagnosis of intussusception of the chronic type, probably starting in the caput cæci. Needless to say, I was immensely pleased when my diagnosis proved correct. It was based on the occurrence of sudden colic in a healthy child associated with a variability in the resistance of the tumour, proving it to be one of the bowel.

**Case 4.**—On August 27th, 1927, a baby aged thirteen months was seized with acute abdominal pain; it screamed all day long and passed several loose motions. Following this it became constipated, but aperients produced several loose actions. Vomiting and attacks of pain continued with intermissions. Eleven days later it was admitted to the Children's Hospital on the medical side. That day an enema produced a loose motion. The child vomited once. Its weight that day (September 7th) was noted to be three pounds less than it had been in the previous June. Two days later all the original symptoms recurred, and for the first time it passed blood per rectum. I was asked to see the child. The right iliac fossa was empty. In the left iliac fossa was a sausage-shaped tumour of variable consistency, and the diagnosis was perfectly obvious. I operated, and was able quite easily to reduce an intussusception, which had started at the caput cæci and had travelled round to the sigmoid flexure.

Quite a large number of cases of chronic intussusception have been published, but the condition is by no means a common one, nor is the possibility of its occurrence sufficiently widely recognised. The main features are: (1) sudden onset of very acute abdominal pain, with vomiting; (2) alternating diarrhœa and constipation, the motions sometimes containing blood; (3) rapid wasting; (4) periods of intermission of symptoms; (5) an abdominal tumour. The rapid wasting, irregularity of the bowels, occasional blood in the motions, abdominal distention, and a transverse abdominal tumour have led to the diagnosis of tuberculous peritonitis on several occasions, and in one of these the diagnosis was only established post mortem.

An ordinary intussusception has rarely to be distinguished from any other complaint except acute enteritis and purpura. In the former the onset is less acute, and is accompanied by diarrhœa. The motions are offensive (in intussusception they are not) and fæces and blood are mixed together. No tumour is present.

In purpura the diagnosis is impossible when hemorrhage into the bowel wall has produced a tumour, unless purpuric patches are present elsewhere in the body. It is suggested if the fæces contain blood but no mucus. The age is often an unusual one for intussusception, and there is no emptiness in the right iliac fossa.

In all these cases treatment has been operative. A small incision, big enough to admit two fingers, has been made. The finger-tips have been placed at the back of the apex of the intussusception, the left hand has been placed on the abdominal wall, and between the finger-tips and the hand the intussusception has been gradually pushed back.

The last inch or two alone presents any difficulty. This often has to be pulled out of the wound in order that the œdema may be slowly and steadily squeezed out before reduction is possible. The essential thing is to be gentle and take time. The necessity for performing an enterectomy never arises in early cases. The mortality arising from it is so appalling that I have determined not to do any more primary enterectomies, but simply to pull out the gangrenous gut, drain the bowel above, and do my enterectomy a few days later, thus bringing the treatment into line with that of other late cases of intestinal obstruction.

Reduction by means of rectal injection has always been unsatisfactory up to the present, on account of the difficulty in determining whether reduction had taken place or not. Were all cases diagnosed and treated sufficiently early, the mortality would be negligible. Sir William Taylor[1] lost three only out of a series of eighty-one cases, but the statistics of seven London and provincial hospitals[2] show that the average mortality for the five years, 1920-24, amounted to 22 per cent.

### Discussion.

Mr. A. R. Short pointed out that the recent fall in mortality must be ascribed to early operation. The local incidence of intussusception was below the average. He mentioned the unwisdom of early removal of the stitches after operation. Hipsley's method of inflation of the bowel had given the lowest recorded mortality of any large series of cases.

Mr. C. F. Walters inquired whether the presence of bile in the fæces was of definite diagnostic significance. He mentioned two cases of chronic intussusception in adults in his own experience.

Mr. A. W. Adams suggested that radiography of an opaque enema might be of value in diagnosis, and referred to cases of undoubted intussusception that had undergone spontaneous reduction.

Dr. H. Rogers referred to the familiar predisposition to intussusception. He had seen three children in a family of four die of it.

Dr. C. A. H. Gee mentioned two cases occurring in one family. Apropos of Mr. Short's warning, in both of these the wound burst open subsequently. However, they seemed none the worse for it.

MR. DUNCAN WOOD mentioned the occurrence of three cases in one family with two deaths.

DR. NEWMAN NEILD thought that the fall in mortality was due to early diagnosis and early calling in of a surgeon. Cases were not now, as formerly, treated at first as medical.

MR. CHITTY replied.

REFERENCES

[1] *Brit. M. J.*, Nov., 1925, p. 993.
[2] *Ibid.*

